# The association between the gut microbiota metabolite trimethylamine N-oxide and heart failure

**DOI:** 10.3389/fmicb.2024.1440241

**Published:** 2024-09-26

**Authors:** Zharkyn Jarmukhanov, Nurislam Mukhanbetzhanov, Samat Kozhakhmetov, Madiyar Nurgaziyev, Aliya Sailybayeva, Makhabbat Bekbossynova, Almagul Kushugulova

**Affiliations:** ^1^Laboratory of Microbiome, Center for Life Sciences, National Laboratory Astana, Nazarbayev University, Astana, Kazakhstan; ^2^Heart Center, CF “University Medical Center”, Astana, Kazakhstan

**Keywords:** TMAO, heart failure, gut microbiome, metabolite, chronic heart failure

## Abstract

This systematic review explores the relationship between the gut microbiota metabolite trimethylamine N-oxide (TMAO) and heart failure (HF), given the significant impact of TMAO on cardiovascular health. A systematic search and meta-analysis of peer-reviewed studies published from 2013 to 2024 were conducted, focusing on adult patients with heart failure and healthy controls. The review found that elevated levels of TMAO are associated with atherosclerosis, endothelial dysfunction, and increased cardiovascular disease risk, all of which can exacerbate heart failure. The analysis also highlights that high TMAO levels are linked to reduced left ventricular ejection fraction (LVEF) and glomerular filtration rate (GFR), further supporting TMAO’s role as a biomarker in heart failure assessment. The findings suggest that interventions targeting gut microbiota to reduce TMAO could potentially benefit patients with heart failure, although further research is needed to evaluate the effectiveness of such approaches.

## 1 Introduction

Heart failure (HF) is a major global healthcare burden due to its high prevalence and unfavorable prognosis. Heart failure represents the terminal stage of different forms of cardiovascular illness. Recent findings have also emphasized the potential significance of modified gut microbiota and its byproducts in the development and outcome of HF (Suzuki et al., 2016; Tang et al., 2014; Trøseid et al., 2015). Studies have demonstrated that various microbial metabolites, including peptidoglycan and lipopolysaccharide, continuously translocate into the portal circulation (Clarke et al., 2010; Balmer et al., 2014). According to the gut hypothesis of HF, decreased cardiac output and impaired systemic circulation can cause intestinal hypoperfusion and mucosal ischemia. As a result, the intestinal barrier function is impaired, which increases its permeability and facilitates the penetration of microorganisms and their metabolites into the systemic circulation. This can lead to the development of chronic low-grade inflammation in patients with HF (Chioncel and Ambrosy, 2019).

Trimethylamine (TMA) is a nitrogenous compound that performs essential functions in physiological mechanisms across various organisms. TMA, originating from choline, carnitine, or betaine within the intestinal tract, undergoes conversion to trimethylamine N-oxide through the action of liver enzyme flavin-dependent monooxygenase 3. TMAO, or trimethylamine N-oxide, has emerged as an established cardiovascular and metabolic risk factor (Shafi et al., 2017). While reviewed, it has also been discovered that the mechanism by which TMAO affects the cardiovascular system may also be related to its atherosclerosis-promoting and inflammation induction roles (Suzuki et al., 2016). In clinical studies, it was revealed that elevated TMAO may have a strong relationship with heart failure, which can be summarized as the result of a proatherogenic role of TMAO and sequential advancement of inflammation in the bloodstream (Suzuki et al., 2016). In addition, as seen from the pathogenesis and discussed topics above, TMAO can also cause endothelial dysfunction, which is of significance when determining the drug’s role in the treatment of heart failure or possibly preventive strategies. Therefore, the relationship between TMAO and heart failure may also help to understand the mechanism itself and identify TMAO as a potential preventive mechanism in the future.

## 2 Materials and methods

### 2.1 Search strategy and eligibility criteria

The search was conducted following the guidelines of the 2020 Preferred Reporting Items for Systematic Reviews and Meta-Analyses guideline (Page et al., 2021). Two authors independently conducted a systematic search of the available peer-reviewed papers published from 2013 until 2024, using three online databases, including MEDLINE, EMBASE, and PUBMED. We developed a search strategy, using “gut microbiota,” “heart failure” or “hearth insufficiency,” and “TMAO,” and their combinations in association with keywords and synonyms. The subject of analysis was human research that analyzed TMAO concentration and gut microbiota profile in adult patients affected by HF and in healthy controls, over 18 years. We included only observational studies, while intervention studies related to oncological disease were first excluded using relevance. A more detailed search strategy was provided as Supplementary Table 2.

### 2.2 Study collection and data retrieval

**Figure 1:**
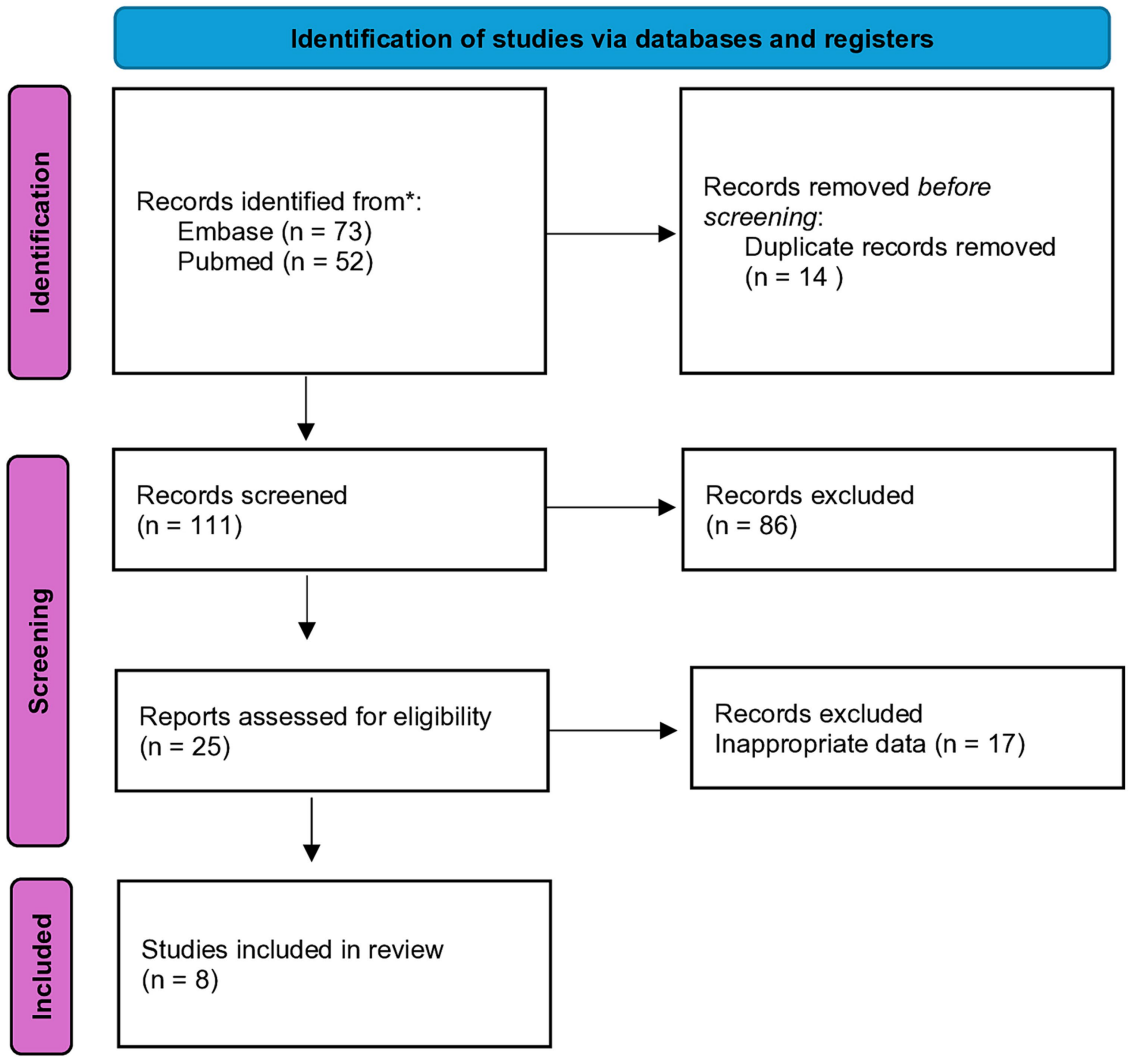
Flow chart of the literature search and study selection process.

The articles sourced from online databases were imported into Endnote 21 to facilitate the selection of relevant studies. A total of 125 studies were identified, and after removing duplicates, 111 articles remained for screening. From the screened articles, 8 full-text papers were assessed for eligibility (Figure 1). A structured form for data extraction was used to collect important details from the studies. This encompassed details about the author and release date, research site, structure, initial traits of subjects with heart failure, and healthy controls (e.g., group size, age, gender, body mass index, hypertension incidence, and diabetes prevalence among others), specifics regarding outcome assessment (including methods for sample collection and processing beyond microbiome analysis), analytical process, and reference database.

### 2.3 Data analysis

To estimate the combined effects using RRs and 95% CI, the data were processed using Revman (version 5.4.1). The *Z*-test was used to estimate the overall effect, and *p* < 0.05 (2-tailed) was considered statistically significant. We evaluated potential heterogeneity with I2 statistics. This analysis incorporated outcomes as continuous variables, utilizing their mean and standard deviation (SD). Mean differences were specifically applied to various health indicators, such as age, BMI, gender, smoking status, LV ejection fraction, hypertension, diabetes mellitus etc. A structured subgroup analysis of the influence of various factors on heart failure outcomes according to TMAO levels was performed using various statistical measures and is shown in Table 1. Circulating levels of TMAO were assessed in non-fasting venous blood samples using stable isotope dilution liquid chromatography–tandem mass spectrometry (LC–MS/MS).

**Table 1:** Subgroup analysis of the association circulating TMAO concentrations for heart failure according to study characteristics.

Name	Studies, *n*	Effect measure	Analysis model	Effect estimate	*p* value	*I*^2^, %	*p* value
Age	5 (Amrein et al., 2022; Emoto et al., 2021; Kinugasa et al., 2021; Tang et al., 2014; Zong et al., 2022)	Mean difference	Fixed	−3.0 (−3.8, −2,2)	0.27	21	0
BMI	3 (Amrein et al., 2022; Kinugasa et al., 2021; Zong et al., 2022)	Mean difference	Random	−0.14 (−1.04, 0.75)	0.01	76	0.75
Male	5 (Amrein et al., 2022; Emoto et al., 2021; Kinugasa et al., 2021; Tang et al., 2014; Zong et al., 2022)	Risk ratio	Random	0.8 (0.6, 0.9)	0.0001	82	0.01
Smoking	3 (Amrein et al., 2022; Emoto et al., 2021; Zong et al., 2022)	Risk ratio	Random	0.89 (0.83, 0.96)	0.7	0	0.002
LV ejection fraction, %	5 (Amrein et al., 2022; Emoto et al., 2021; Kinugasa et al., 2021; Zong et al., 2022)	Mean difference	Random	4.62 (2.62, 6.62)	0.014	45	0.000
Hypertension, %	5 (Amrein et al., 2022; Emoto et al., 2021; Kinugasa et al., 2021; Tang et al., 2014; Zong et al., 2022)	Risk ratio	Fixed	0.92 (0.89, 0.95)	0.2	32	0.00
Diabetes mellitus	4 (Emoto et al., 2021; Kinugasa et al., 2021; Tang et al., 2014; Zong et al., 2022)	Risk ratio	Random	0.85 (0.58, 1.24)	0.003	78	0.40
Dyslipidemia	3 (Emoto et al., 2021; Kinugasa et al., 2021; Zong et al., 2022)	Risk ratio	Random	1.11 (0.86, 1.42)	0.5	0	0.39
Atrial fibrillation	2 (Emoto et al., 2021; Kinugasa et al., 2021)	Risk ratio	Fixed	1.01 (0.76, 1.35)	0.8	0	0.9
Creatinine, mg/dl	2 (Emoto et al., 2021; Zong et al., 2022)	Mean difference	Random	−17.54 (−52.5, 17.4)	0.000	96	0.32
eGFR, ml/mm/1.73 m2	5 (Amrein et al., 2022; Emoto et al., 2021; Kinugasa et al., 2021; Tang et al., 2014; Zong et al., 2022)	Mean difference	Random	16.21 (8.31, 24.11)	0.000	94	0.00
BNP, pg/ml	3 (Emoto et al., 2021; Kinugasa et al., 2021; Tang et al., 2014)	Mean difference	Random	−100.7 (−242.4, 40.96)	0.002	83	0.16

## 3 Results

### 3.1 Study characteristics and quality assessments

Table 2 provides a summary of the study populations and specific characteristics of the included research. Eight full-text publications in all completed the requirements for assessing the gut microbiota profiles and TMAO concentrations in adult heart failure patients. Based on the methodology, sample size, and study design, the papers were evaluated for quality. Across the eight included studies are the average circulation values of TMAO varied from 1.2 to 38.34 μM.

**Table 2:** Patients characteristics.

First author	Country	Study period	Case/control	*N*	Age	Male (%)	TMAO (Average)	NOS
Amrein et al. (2022)	Switzerland	2010–2016 year	History of heart failure	54	77	1,133 (65.6)	58 4.7 (3.1, 7.2) have fCAD	8
							25 5.3 (3.5, 8.8) do not have fCAD	
Hayashi et al. (2018)	Japan	October 2016 and April 2017	Decomp HF	22	72 ± 18	14 (64%)	NA	8
			Control	11	72 ± 7	6 (55%)		
			Comp HF	22	N/A	N/A		
Emoto et al. (2021)	Japan		Control (Low TMAO)	7	69	4 (57%)	5.2 (3.8–6.7)	7
			Control (High TMAO)	4	76	2 (50%)	11.5 (11.0–17.3)	
			HF (Low TMAO)	9	70	7 (78%)	6.4 (4.5–8.6)	
			HF (High TMAO)	13	79	7 (54%)	21.0 (17.0–34.5)	
Tang et al. (2014)	USA	2001–2007 year	TMAO <5	59	64 ± 11	59%	3 (2.2–4)	5
			μM TMAO ≥5 μM	59	68 ± 11	59%	8.5 (6.6–13.6)	
Trøseid et al. (2015)	Norway		Control	76	66.5 ± 7.1	NA	NA	7
Kinugasa et al. (2021)	Japan	2012–2017	Low TMAO	73	71–83	NA	10,39 (6,92-15,58)	7
			High TMAO	73	75–86		38,34 (27,16-57,79)	
Dong et al. (2021)	China	2016–2017	HfpEF	61	63.23(13.83)	25(41%)	6.84 (1.12)	
			Control	57	61.91(9.58)	23(40.3%)	1.63 (0.08)	
Zong et al. (2022)	China	NA	0.54 μM ≤ TML < 0.75 μM	319	61.3 ± 11.7	226(70.8)	1.2 ± 1.5	
			TML ≥ 0.75 μM	319	62.7 ± 11.4	239(74.9)	2.2 ± 4.1	

Data are *n* (%); mean ± SD; or median [IQR] or {range}; CHF, chronic heart failure; HC, healthy control; HFpEF, heart failure with preserved ejection fraction; HFrEF, heart failure with reduced ejection fraction; N, number of subjects; N/A, not applicable; NOS, Newcastle-Ottawa Scale score; NR, not reported; TMAO, trimethylamine N-oxide; fCAD, functionally relevant coronary artery disease; Decomp HF, decompensated heart failure; Comp HF, compensated heart failure.

The impact of circulating TMAO on patients with heart failurewhich includes ischemic heart disease (Yazaki et al., 2020), diabetes mellitus (Hayashi et al., 2018; Kinugasa et al., 2021; Tang et al., 2014; Yazaki et al., 2020), hypertension (Hayashi et al., 2018; Yazaki et al., 2020), atrial fibrillation (Emoto et al., 2021; Kinugasa et al., 2021), and a number of other comorbidities was the main focus of the research examined in this systematic review.

In total, three studies were conducted in Japan (Emoto et al., 2021; Hayashi et al., 2018; Kinugasa et al., 2021), two in Europe (Amrein et al., 2022; Trøseid et al., 2015), two in China (Dong et al., 2021; Zong et al., 2022) and one in the United States (Tang et al., 2014).

Study quality was high in most of the included cohort studies, with an average NOS score of 6.9 points.

### 3.2 Gut microbiota composition

Recent studies on the gut microbiota in individuals with heart failure have revealed a change in the gut microbial community compared to healthy individuals or those with different comorbidities, medications, and diets than those of heart failure patients (Kamo et al., 2017; Luedde et al., 2017). Researching the specific microbial species and pathways involved in TMAO production may reveal possible microbial targets to regulate TMAO levels and reduce the risk of heart failure.

Out of the eight articles reviewed, only two conducted comparisons of gut microbiome composition between patients clinically diagnosed with heart failure and control groups. A research investigation noted an increase in the presence of the *Actinobacteria* phylum and *Bifidobacterium* genus, as well as a decrease in the abundance of the *Megamonas* genus in patients with heart failure compared to control subjects using 16S rRNA gene amplicon sequencing (Hayashi et al., 2018). Moreover, a study found that the *Escherichia/Shigella* genus was more prevalent in decompensated heart failure than in compensated phase HF within the same patient (Hayashi et al., 2018).

Another study discovered a positive correlation between the existence of cntA/B and TMAO, especially in individuals with HF. The presence of cntA/B was mainly observed in Escherichia and Klebsiella bacteria among both control subjects and individuals with HF (Emoto et al., 2021).

### 3.3 Meta-analysis of the correlations between circulating TMAO concentrations and left ventricular ejection fraction

**Figure 2:**
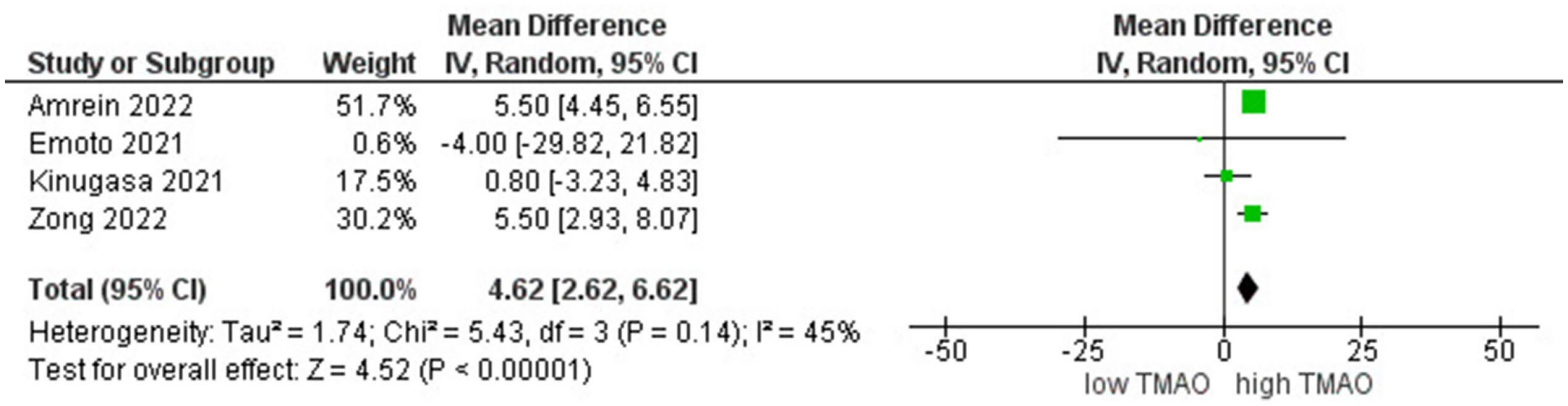
A forest plot of correlations between TMAO and LVEF.

We included 5 studies involving 3,300 individuals to perform the meta-analyses of correlations between TMAO and LVEF. Results suggested that circulating TMAO concentrations are strongly correlated with LVEF in high TMAO level patients (*r* = 4.62; 95% CI = 2.62, 6.62; *p* < 0.000006, Figure 2).

### 3.4 Correlation between TMAO concentrations and eGFR

**Figure 3:**
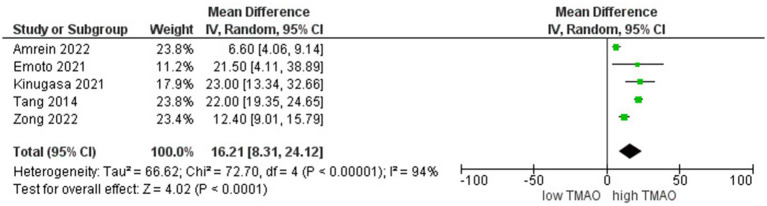
A forest plot of correlations between TMAO and eGFR.

A comparative study of individuals with different levels of circulating TMAO concentrations involved 3,300 participants. We found a stronger inverse correlation between high levels of TMAO and eGFR (*r* = 16.2; 95% CI = 8.3, 24.11; *p* < 0.00005, Figure 3).

An organized meta-analysis examining the impact of different variables on heart failure results based on TMAO levels, utilizing statistical methods such as mean differences and risk ratios. The examination covers various categories such as age, BMI, sex, smoking habits, LV ejection fraction, high blood pressure, diabetes, high cholesterol, atrial fibrillation, creatinine levels, eGFR, and BNP levels. The research uses fixed and random effects models to account for the anticipated variations among studies. The *p*-values given show statistical significance, such as 0.000006 for LV ejection fraction highlighting robust connections. The *I*^2^% values indicate how much diversity exists among the studies included, with high values indicating significant variation that could affect the understanding of the findings.

## 4 Discussion

*Tang* and colleagues introduced the concept of the “gut hypothesis of heart failure,” which posits that reduced cardiac output in heart failure can cause reduced blood circulation to the intestines, resulting in damage to the intestinal mucosa. This dysfunction in the lining of the intestines can lead to increased permeability, poor nutrition, bacteria passing through the intestinal wall, and higher circulating levels of endotoxins, which may be connected to inflammation in heart failure (Nagatomo and Wilson Tang, 2015). Previous research showed that increased SCFA production capacity within the synbiotic group was associated with significant reductions in diastolic blood pressure (Bartolomaeus et al., 2020).

TMAO is formed by the liver enzyme flavin monooxygenase from TMA, which comes from bacterial TMA lyases in the gut breaking down dietary phosphatidylcholine, choline, and carnitine (Organ et al., 2016). Clinical and experimental data indicate potential involvement of choline and TMAO in the progression of HF (Organ et al., 2016; Suzuki et al., 2016; Tang et al., 2014). Different types of bacteria, including *Escherichia fergusonii, Clostridium sporogenes*, *Edwardsiella tarda, Anaerococcus hydrogenalis, Clostridium asparagiforme, Clostridium hathewayi, Providencia rettgeri* and *Proteus penneri*, have been found in the human gut producing TMA from choline (Romano et al., 2015). Additionally, alterations in the ratio of Bacteroidetes to Firmicutes were noticed in young, healthy men with elevated TMAO levels following the consumption of TMA precursors. This indicates that TMAO production is influenced by the level of gut microbial activity (Cho et al., 2017). Distinct differences were found in the gut bacteria makeup of CHF patients versus healthy individuals, suggesting a possible link between CHF and imbalances in intestinal flora. *Firmicutes* and *Bacteroides* are the main phyla found in the microbiome of healthy intestines. These groups are closely linked to the environment, impacting human and animal health in both positive and negative ways. Nonetheless, a significant decrease in *Firmicutes* levels was observed in severe CHF patients in this research (Sun et al., 2022). Previous research showed that patients with heart failure have an excess of harmful bacteria in their intestines such as *Shigella, Campylobacter*, and *Salmonella*, which varies based on the severity of their heart failure (Pasini et al., 2016). In line with these results, the study discovered an increased presence of *Escherichia/Shigella* during the decompensated stage of HF compared to the compensated stage in the same patient, utilizing non-culture-based techniques. It is interesting that the high presence of *Escherichia/Shigella* was not only linked with increased levels of TMAO and IS in the bloodstream but also included bacteria that produce indole with tryptophanases (Kanehisa et al., 2017; Tatusov et al., 2003). Kiouptsi et al. explored the role of the gut microbiota in the expression of protein disulfide isomerase (PDI) and PDIA6 under hypoxic conditions and their subsequent effects on cardiac tissue. Their findings revealed that, in the absence of gut microbiota, there is a significant decrease in PDIA6 expression. This underscores the importance of the gut microbiome in regulating the unfolded protein response (UPR) mechanisms during myocardial infarction (Kiouptsi and Reinhardt, 2018).

Multiple research studies have shown that the breakdown of dietary carnitine by gut bacteria leads to the creation of TMAO, which is linked to the development of heart failure (Koeth et al., 2013). The relationship among diet, gut bacteria, and metabolite production like TMAO and IS highlights the possible influence of the gut-heart connection on the advancement and seriousness of heart failure.

People with heart failure can be grouped into three categories based on their left ventricular ejection fraction (LVEF): heart failure with reduced ejection fraction (HFrEF) for LVEF below 40%; heart failure with mildly reduced ejection fraction (HFmrEF) for LVEF ranging from 40% to less than 50%; and heart failure with preserved ejection fraction (HFpEF) for LVEF equal to or greater than 50% (McDonagh et al., 2021). This parameter, obtained via transthoracic echocardiography, offers important details regarding the left ventricle’s role in ejecting blood from the heart. It is crucial to monitor the left ventricular ejection fraction to evaluate cardiac function and assess the severity of heart disease. An ejection fraction within the typical range is 50–70%, while lower values suggest a reduced pumping capacity of the heart.

TMAO is responsible for disrupting mitochondrial function, leading to an increase in oxidative stress and endothelial dysfunction (Zheng and He, 2022). These are critical factors in heart failure pathology.

Endothelial dysfunction, characterized by impaired vasodilation, is closely associated with oxidative stress and heightened inflammatory processes. These processes involve the formation of foam cells, production of inflammatory cytokines (Marshall et al., 2017; Ng et al., 2017), all of which influence blood clotting, immune responses, and regulation of vascular tone (Marshall et al., 2017). TMAO is thought to contribute to endothelial dysfunction by reducing endothelial cell viability, increasing reactive oxygen species, enhancing vascular inflammation and calcification, and impairing vascular tone. These factors have been implicated in the development of cardiovascular and cardiometabolic diseases. Trimethylamine N-oxide (TMAO) plays a significant role in contributing to and exacerbating systemic inflammation, which, in turn, impacts cardiovascular health. TMAO activates various inflammatory signaling pathways. This activation leads to the increased production of pro-inflammatory cytokines such as interleukin-1β (IL-1β), tumor necrosis factor-alpha (TNF-*α*), and IL-6 (Zhang and An, 2007). TMAO stimulates the production of pro-inflammatory cytokines, including TNF-α and IL-1β (Chen et al., 2017; Boutagy et al., 2015), by activating the nuclear factor-κB (NF-κB) signaling pathway in endothelial cells (Seldin et al., 2016). This activation promotes leukocyte adhesion to the endothelial walls, leading to endothelial dysfunction, which increases the risk of cardiovascular diseases such as thrombosis and atherosclerosis (Yang et al., 2019).

In addition to affecting endothelial function, TMAO increases oxidative stress by enhancing the production of reactive oxygen species (ROS). Many studies have demonstrated that high TMAO concentrations induce endothelial dysfunction in cultured endothelial cells through oxidative stress (Singh et al., 2019; Li et al., 2017). Specifically, TMAO has been shown to trigger ROS production through thioredoxin-interacting protein- NOD-, LRR- and pyrin domain-containing protein 3 (TXNIP-NLRP3). It was demonstrated that the TXNIP-NLRP3 inflammasome complex production was activated in a time and dose-dependent manner by TMAO (Sun et al., 2016). Besides TMAO has been shown to activate enzymes like NADPH oxidase, leading to elevated ROS levels and oxidative damage to endothelial cells. This oxidative stress further promotes inflammation by activating redox-sensitive signaling pathways involved in cardiovascular disease pathogenesis (González-Loyola and Petrova, 2021).

TMAO has been found to worsen atherosclerosis by increasing cholesterol accumulation in macrophages and causing the formation of foam cells (Ding et al., 2018). This process is crucial in the progression of cardiovascular diseases, including heart failure. Foam cells are created when macrophages absorb excessive cholesterol from lipoproteins via transporters such as CD36, SR-A, and LOX-1. As these macrophages become overloaded with cholesterol, they transform into foam cells, which then accumulate in the walls of blood vessels, contributing to the progression of atherosclerosis (Zheng et al., 2016; Gui et al., 2022; Bentzon et al., 2014).

Endothelial dysfunction is linked to abnormal vascular tone due to imbalances in the production of three key vasoactive factors: nitric oxide (NO), prostaglandin I2 (PGI2), and endothelium-derived hyperpolarization (EDH). These factors work together to regulate vascular tone by promoting vasodilation, inhibiting platelet aggregation and smooth muscle cell proliferation, and inducing muscle relaxation, and disruptions in their balance contribute to endothelial dysfunction (Matsumoto et al., 2020; Leo et al., 2017).

Studies also suggest that TMAO can directly impact cardiac function by changing calcium handling and myocardial contractility (Oakley et al., 2020). By understanding these pathways in greater detail, we may be able to find new targets for therapeutic intervention that could alleviate heart failure severity in patients with high TMAO levels. These mechanistic pathways highlight the complex relationship between metabolic products of the gut microbiome and cardiovascular health, emphasizing the potential for targeted interventions that modify TMAO levels. For example, recent research demonstrates that increased TMAO levels can exacerbate conditions associated with heart and kidney function (Li et al., 2023). High levels of TMAO in heart failure patients are significantly associated with adverse outcomes and can indicate a worse prognosis (Kinugasa et al., 2021). Particularly, in the context of cardiorenal syndrome, TMAO is not only a marker but also a simple to the pathogenesis factor (Zhang et al., 2023). In this situation, patients with heart failure with preserved LVEF and high levels of TMAO measured indicated that increased TMAO was associated with a poor outcome of hospitalization and death (Kinugasa et al., 2021). Emerging evidence has established that elevated plasma TMAO levels serve as a predictor of cardiovascular disease risk and have been recognized as a valuable biomarker for assessing this risk. In a study involving 720 patients with stable chronic heart failure, heightened TMAO concentrations were shown for the first time to be associated with an increased risk of cardiovascular events. Compared to age- and sex-matched individuals without heart failure, those with chronic heart failure exhibited significantly higher TMAO levels, which were linked to a 3.4-fold increase in mortality risk. Importantly, this elevated risk persisted even after adjusting for traditional risk factors and cardiorenal indices, indicating that high TMAO levels can independently forecast a greater risk of five-year mortality (Tang et al., 2014). Furthermore, a meta-analysis conducted by Schiattarella et al. (2017) corroborated that elevated plasma TMAO levels significantly increased the risk of major adverse cardiovascular events and cerebrovascular events by 67%, as well as all-cause mortality by 91%. Additionally, for every 10 μmol/L rise in plasma TMAO concentration, the risk of all-cause mortality increased by 7.6%. Another meta-analysis revealed that among patients with chronic heart conditions, high TMAO levels raised the risk of major adverse cardiovascular events by 58%, with an even more pronounced risk observed in individuals with a longer follow-up period of 4 years or more (Yao et al., 2020). Suzuki et al. (2016) found that circulating TMAO is a marker for predicting mortality and heart failure (HF) within 1 year in acute heart failure (AHF) patients. However, its predictive power diminished after adjusting for renal function, suggesting a link between TMAO levels and renal parameters. Combining TMAO with other markers, such as NT-proBNP, improved risk assessment, especially in Caucasian patients, but more extensive clinical studies are needed to validate these findings across different populations (Yazaki et al., 2020).

Other studies, on the other hand, show that there is a more complex relationship, and aspects such as TMAO may not be the only indicator of the outcome of heart failure (Jaworska et al., 2019). Comparing these studies allows researchers to distinguish the conditions in which the associations between this biomarker and adverse outcomes are most pronounced and can be used to design more personalized methods of managing heart failure. Therefore, the current comparative analysis not only confirms this biomarker’s clinical significance but also indicates the complexity of assessing its role in cardiovascular health and the need for a comprehensive evaluation in clinical settings.

The potential therapeutic effects of prebiotics and probiotics in modulating gut microbiota composition and controlling TMAO levels are discussed (Zhang et al., 2015). Prebiotics, such as galactooligosaccharides, fructooligosaccharides, inulin, and dietary fibers, serve as fermentable substrates that are metabolized by the host’s gut microbiota (Silva et al., 2021; Swanson et al., 2020). Specific probiotics such as *Lactobacillus paracasei* have been shown to reduce TMA formation in animal models, and other strains like *Lactobacillus* and *Bifidobacterium* are associated with a reduced risk of atherosclerosis (Ma and Li, 2018; Martin et al., 2008). Methanogenic bacteria, such as Methanomassiliicoccus luminyensis B10, have also been used to metabolize and deplete TMA (Dridi, 2012; Brugère et al., 2014).

Probiotics can regulate inflammatory pathways, reduce intestinal inflammation, and impact cytokine production and signaling (Pourrajab et al., 2020; Yousefi et al., 2019). However, their effectiveness may be limited by the diversity of the host’s gut microbiome.

Antibiotics can reduce TMAO levels by changing the gut microbiome. However, antibiotics also have risks, such as temporary TMAO fluctuations, potential antibiotic resistance, and other safety issues (Winkel et al., 2015). For instance, extended antibiotic use in older women has been linked to increased inflammation due to gut microbiome changes, suggesting the need for careful antibiotic use (Heianza et al., 2019).

Therefore, utilizing TMAO level testing as a common diagnostic retrial would enable the early diagnosis and management of this heart condition and promote patient-specific starting. In turn, it makes it likely to choose regular interventions before the condition deteriorates Clinical signs. Further, TMAO testing would help improve on patients’ diets an d recommend treatment, including that of using approved probiotics or standard pharmacological agents such as 3,3-dipropylindum carbocyanine iodide to alter the gut microbiota and lower TMAO levels. It would also enhance the selection process for individuals eligible for further observation, as TMAO testing could be a crucial stratification mark in clinical assessments due to its usefulness in determining the various patient response dynamics. Long-term monitoring for TMAO levels is also pivotal since it would enable the identification of cases of significant renal decline in heart failure patients. Overall, this information would significantly improve the mortality rates associated with heart failure.

## 5 Conclusion

To sum up, the present systematic review has demonstrated a robust relationship between elevated levels of trimethylamine N-oxide and unfavorable cardiovascular events, particularly in heart failure. Moreover, evidence on the correlation between high TMAO levels and left ventricular ejection fraction and glomerular filtration rate demonstrated the potential of TMAO level as a biomarker to evaluate the severity and progression of HF. Finally, considering the substantial relationship between gut microbiota with TMAO production, it might be appropriate to include the strategies to modulate the gut microbiome. In general, the findings of this systematic review proved the significance of TMAO in HF as a potential biomarker for prognosis and treatment.

## 6 Study limitations

Of the eight articles included in the review, only two provided data on the gut microbiota composition in heart failure. This insufficient representation restricts the ability to comprehensively analyze the interaction between gut microbiota, TMAO levels, and heart failure. The lack of detailed microbiota data could hinder the interpretation of how specific microbiota changes contribute to variations in TMAO levels and influence heart failure outcomes.

## Data Availability

The original contributions presented in the study are included in the article/Supplementary material, further inquiries can be directed to the corresponding author.

## References

[ref1] Amrein M. Li X. S. Walter J. Wang Z. Zimmermann T. Strebel I. . (2022). Gut microbiota-dependent metabolite trimethylamine N-oxide (TMAO) and cardiovascular risk in patients with suspected functionally relevant coronary artery disease (fCAD). Clin. Res. Cardiol. 111, 692–704. doi: 10.1007/s00392-022-01992-6, PMID: 35220448 PMC9151506

[ref2] Balmer M. L. Slack E. de Gottardi A. Lawson M. A. E. Hapfelmeier S. Miele L. . (2014). The liver may act as a firewall mediating mutualism between the host and its gut commensal microbiota. Sci. Transl. Med. 6:237ra66. doi: 10.1126/scitranslmed.3008618, PMID: 24848256

[ref3] Bartolomaeus H. Avery E. G. Bartolomaeus T. U. P. Kozhakhmetov S. Zhumadilov Z. Müller D. N. . (2020). Blood pressure changes correlate with short-chain fatty acid production potential shifts under a Synbiotic intervention. Cardiovasc. Res. 116, 1252–1253. doi: 10.1093/cvr/cvaa083, PMID: 32232443

[ref4] Bentzon J. F. Otsuka F. Virmani R. Falk E. (2014). Mechanisms of plaque formation and rupture. Circ. Res. 114, 1852–1866. doi: 10.1161/CIRCRESAHA.114.302721 24902970

[ref5] Boutagy N. E. Neilson A. P. Osterberg K. L. Smithson A. T. Englund T. R. Davy B. M. . (2015). Probiotic supplementation and trimethylamine- *N* -oxide production following a high-fat diet. Obesity 23, 2357–2363. doi: 10.1002/oby.21212, PMID: 26465927

[ref6] Brugère J.-F. Borrel G. Gaci N. Tottey W. O’Toole P. W. Malpuech-Brugère C. (2014). Archaebiotics: proposed therapeutic use of Archaea to prevent Trimethylaminuria and cardiovascular disease. Gut Microbes 5, 5–10. doi: 10.4161/gmic.26749, PMID: 24247281 PMC4049937

[ref7] Chen M.-l. Zhu X.-h. Ran L. Lang H.-d. Yi L. Mi M.-t. (2017). Trimethylamine-N-oxide induces vascular inflammation by activating the NLRP3 Inflammasome through the SIRT3-SOD2-mtROS signaling pathway. J. Am. Heart Assoc. 6:e006347. doi: 10.1161/JAHA.117.006347, PMID: 28871042 PMC5634285

[ref8] Chioncel O. Ambrosy A. P. (2019). Trimethylamine N-oxide and risk of heart failure progression: marker or mediator of disease. Eur. J. Heart Fail. 21, 887–890. doi: 10.1002/ejhf.1409 30623560

[ref9] Cho C. E. Taesuwan S. Malysheva O. V. Bender E. Tulchinsky N. F. Yan J. . (2017). Trimethylamine- *N* -oxide (TMAO) response to animal source foods varies among healthy young men and is influenced by their gut microbiota composition: a randomized controlled trial. Mol. Nutr. Food Res. 61:1600324. doi: 10.1002/mnfr.201600324, PMID: 27377678

[ref10] Clarke T. B. Davis K. M. Lysenko E. S. Zhou A. Y. Yimin Y. Weiser J. N. (2010). Recognition of peptidoglycan from the microbiota by Nod1 enhances systemic innate immunity. Nat. Med. 16, 228–231. doi: 10.1038/nm.2087, PMID: 20081863 PMC4497535

[ref11] Ding L. Chang M. Guo Y. Zhang L. Xue C. Yanagita T. . (2018). Trimethylamine-N-oxide (TMAO)-induced atherosclerosis is associated with bile acid metabolism. Lipids Health Dis. 17:286. doi: 10.1186/s12944-018-0939-6, PMID: 30567573 PMC6300890

[ref12] Dong Z. Zheng S. Shen Z. Luo Y. Hai X. (2021). Trimethylamine N-oxide is associated with heart failure risk in patients with preserved ejection fraction. Lab. Med. 52, 346–351. doi: 10.1093/labmed/lmaa075, PMID: 33135738

[ref13] Dridi B. (2012). Laboratory tools for detection of Archaea in humans. Clin. Microbiol. Infect. 18, 825–833. doi: 10.1111/j.1469-0691.2012.03952.x, PMID: 22897827

[ref14] Emoto T. Hayashi T. Tabata T. Yamashita T. Watanabe H. Takahashi T. . (2021). Metagenomic analysis of gut microbiota reveals its role in trimethylamine metabolism in heart failure. Int. J. Cardiol. 338, 138–142. doi: 10.1016/j.ijcard.2021.06.003, PMID: 34102245

[ref15] González-Loyola A. Petrova T. V. (2021). Development and aging of the lymphatic vascular system. Adv. Drug Deliv. Rev. 169, 63–78. doi: 10.1016/j.addr.2020.12.005, PMID: 33316347

[ref16] Gui Y. Zheng H. Cao R. Y. (2022). Foam cells in atherosclerosis: novel insights into its origins, consequences, and molecular mechanisms. Front Cardiovasc Med 9:845942. doi: 10.3389/fcvm.2022.845942, PMID: 35498045 PMC9043520

[ref17] Hayashi T. Yamashita T. Watanabe H. Kami K. Yoshida N. Tabata T. . (2018). Gut microbiome and plasma microbiome-related metabolites in patients with decompensated and compensated heart failure. Circ. J. 83, 182–192. doi: 10.1253/circj.CJ-18-0468, PMID: 30487369

[ref18] Heianza Y. Zheng Y. Ma W. Rimm E. B. Albert C. M. Hu F. B. . (2019). Duration and life-stage of antibiotic use and risk of cardiovascular events in women. Eur. Heart J. 40, 3838–3845. doi: 10.1093/eurheartj/ehz231, PMID: 31216010 PMC6911167

[ref19] Jaworska K. Hering D. Mosieniak G. Bielak-Zmijewska A. Pilz M. Konwerski M. . (2019). TMA, a forgotten uremic toxin, but not TMAO, is involved in cardiovascular pathology. Toxins 11:490. doi: 10.3390/toxins11090490, PMID: 31454905 PMC6784008

[ref20] Kamo T. Akazawa H. Suda W. Akiko Saga-Kamo Y. Shimizu H. Y. Liu Q. . (2017). Dysbiosis and compositional alterations with aging in the gut microbiota of patients with heart failure. PLOS ONE 12:e0174099. doi: 10.1371/journal.pone.0174099, PMID: 28328981 PMC5362204

[ref21] Kanehisa M. Furumichi M. Tanabe M. Sato Y. Morishima K. (2017). KEGG: new perspectives on genomes, pathways, diseases and drugs. Nucleic Acids Res. 45, D353–D361. doi: 10.1093/nar/gkw1092, PMID: 27899662 PMC5210567

[ref22] Kinugasa Y. Nakamura K. Kamitani H. Hirai M. Yanagihara K. Kato M. . (2021). Trimethylamine N-oxide and outcomes in patients hospitalized with acute heart failure and preserved ejection fraction. ESC Heart Failure 8, 2103–2110. doi: 10.1002/ehf2.13290, PMID: 33734604 PMC8120352

[ref23] Kiouptsi K. Reinhardt C. (2018). Contribution of the commensal microbiota to atherosclerosis and arterial thrombosis. Br. J. Pharmacol. 175, 4439–4449. doi: 10.1111/bph.14483, PMID: 30129122 PMC6255953

[ref24] Koeth R. A. Wang Z. Levison B. S. Buffa J. A. Org E. Sheehy B. T. . (2013). Intestinal microbiota metabolism of L-carnitine, a nutrient in red meat, promotes atherosclerosis. Nat. Med. 19, 576–585. doi: 10.1038/nm.3145, PMID: 23563705 PMC3650111

[ref25] Leo C. H. Fernando D. T. Tran L. Ng H. H. Marshall S. A. Parry L. J. (2017). Serelaxin treatment reduces oxidative stress and increases aldehyde Dehydrogenase-2 to attenuate nitrate tolerance. Front. Pharmacol. 8:141. doi: 10.3389/fphar.2017.00141, PMID: 28377719 PMC5359255

[ref26] Li T. Chen Y. Gua C. Li X. (2017). Elevated circulating trimethylamine N-oxide levels contribute to endothelial dysfunction in aged rats through vascular inflammation and oxidative stress. Front. Physiol. 8:350. doi: 10.3389/fphys.2017.00350, PMID: 28611682 PMC5447752

[ref27] Li Y. Hongmei L. Guo J. Zhang M. Zheng H. Liu Y. . (2023). Gut microbiota-derived trimethylamine N-oxide is associated with the risk of all-cause and cardiovascular mortality in patients with chronic kidney disease: a systematic review and dose-response Meta-analysis. Ann. Med. 55:2215542. doi: 10.1080/07853890.2023.2215542, PMID: 37246850 PMC10228303

[ref28] Luedde M. Winkler T. Heinsen F.-A. Rühlemann M. C. Spehlmann M. E. Bajrovic A. . (2017). Heart failure is associated with depletion of Core intestinal microbiota. ESC Heart Failure 4, 282–290. doi: 10.1002/ehf2.12155, PMID: 28772054 PMC5542738

[ref29] Ma J. Li H. (2018). The role of gut microbiota in atherosclerosis and hypertension. Front. Pharmacol. 9:1082. doi: 10.3389/fphar.2018.01082, PMID: 30319417 PMC6167910

[ref30] Marshall S. A. Leo C. H. Girling J. E. Tare M. Beard S. Hannan N. J. . (2017). Relaxin treatment reduces angiotensin II-induced vasoconstriction in pregnancy and protects against endothelial dysfunction†. Biol. Reprod. 96, 895–906. doi: 10.1093/biolre/iox023, PMID: 28379296

[ref31] Martin F.-P. J. Wang Y. Sprenger N. Yap I. K. S. Lundstedt T. Lek P. . (2008). Probiotic Modulation of Symbiotic Gut Microbial–Host Metabolic Interactions in a Humanized Microbiome Mouse Model. Mol. Syst. Biol. 4:157. doi: 10.1038/msb4100190, PMID: 18197175 PMC2238715

[ref32] Matsumoto T. Kojima M. Takayanagi K. Taguchi K. Kobayashi T. (2020). Role of *S* -Equol, Indoxyl sulfate, and trimethylamine *N* -oxide on vascular function. Am. J. Hypertens. 33, 793–803. doi: 10.1093/ajh/hpaa053, PMID: 32300778 PMC7481967

[ref33] McDonagh T. A. Metra M. Adamo M. Gardner R. S. Baumbach A. Böhm M. . (2021). 2021 ESC guidelines for the diagnosis and treatment of acute and chronic heart failure. Eur. Heart J. 42, 3599–3726. doi: 10.1093/eurheartj/ehab368 34447992

[ref34] Nagatomo Y. Wilson Tang W. H. (2015). Intersections between microbiome and heart failure: revisiting the gut hypothesis. J. Card. Fail. 21, 973–980. doi: 10.1016/j.cardfail.2015.09.017, PMID: 26435097 PMC4666782

[ref35] Ng H. H. Leo C. H. Prakoso D. Qin C. Ritchie R. H. Parry L. J. (2017). Serelaxin treatment reverses vascular dysfunction and left ventricular hypertrophy in a mouse model of type 1 diabetes. Sci. Rep. 7:39604. doi: 10.1038/srep39604, PMID: 28067255 PMC5220363

[ref36] Oakley C. I. Vallejo J. A. Wang D. Gray M. A. Tiede-Lewis L. A. M. Shawgo T. . (2020). Trimethylamine- *N* -oxide acutely increases cardiac muscle contractility. Am. J. Phys. Heart Circ. Phys. 318, H1272–H1282. doi: 10.1152/ajpheart.00507.2019, PMID: 32243768 PMC7346532

[ref37] Organ C. L. Otsuka H. Bhushan S. Wang Z. Bradley J. Trivedi R. . (2016). Choline diet and its gut microbe-derived metabolite, trimethylamine N-oxide, exacerbate pressure overload-induced heart failure. Circ Heart Fail 9:e002314. doi: 10.1161/CIRCHEARTFAILURE.115.002314, PMID: 26699388 PMC4943035

[ref38] Page M. J. McKenzie J. E. Bossuyt P. M. Boutron I. Hoffmann T. C. Mulrow C. D. . (2021). The PRISMA 2020 statement: An updated guideline for reporting systematic reviews. BMJ 21:372:n71. doi: 10.1136/bmj.n71 PMC8005924 33782057

[ref39] Pasini E. Aquilani R. Testa C. Baiardi P. Angioletti S. Boschi F. . (2016). Pathogenic gut Flora in patients with chronic heart failure. JACC Heart Fail 4, 220–227. doi: 10.1016/j.jchf.2015.10.009 26682791

[ref40] Pourrajab B. Fatahi S. Dehnad A. Varkaneh H. K. Shidfar F. (2020). The impact of probiotic yogurt consumption on lipid profiles in subjects with mild to moderate hypercholesterolemia: a systematic review and Meta-analysis of randomized controlled trials. Nutr. Metab. Cardiovasc. Dis. 30, 11–22. doi: 10.1016/j.numecd.2019.10.001, PMID: 31748179

[ref41] Romano K. A. Vivas E. I. Amador-Noguez D. Rey F. E. (2015). Intestinal microbiota composition modulates choline bioavailability from diet and accumulation of the Proatherogenic metabolite trimethylamine- *N* -oxide. mBio 6, e02481–e02414. doi: 10.1128/mBio.02481-14, PMID: 25784704 PMC4453578

[ref42] Schiattarella G. G. Sannino A. Toscano E. Giugliano G. Gargiulo G. Franzone A. . (2017). Gut microbe-generated metabolite trimethylamine-N-oxide as cardiovascular risk biomarker: a systematic review and dose-response Meta-analysis. Eur. Heart J. 38, 2948–2956. doi: 10.1093/eurheartj/ehx342, PMID: 29020409

[ref43] Seldin M. M. Meng Y. Qi H. Zhu W. F. Wang Z. Hazen S. L. . (2016). Trimethylamine N-oxide promotes vascular inflammation through signaling of mitogen-activated protein kinase and nuclear factor-κB. J. Am. Heart Assoc. 5:e002767. doi: 10.1161/JAHA.115.002767, PMID: 26903003 PMC4802459

[ref44] Shafi T. Powe N. R. Meyer T. W. Hwang S. Hai X. Melamed M. L. . (2017). Trimethylamine N-oxide and cardiovascular events in hemodialysis patients. J. Am. Soc. Nephrol. 28, 321–331. doi: 10.1681/ASN.2016030374, PMID: 27436853 PMC5198291

[ref45] Silva D. Ferreira T. Casarotti S. N. De Oliveira G. L. V. Penna A. L. B. (2021). The impact of probiotics, prebiotics, and Synbiotics on the biochemical, clinical, and immunological markers, as well as on the gut microbiota of obese hosts. Crit. Rev. Food Sci. Nutr. 61, 337–355. doi: 10.1080/10408398.2020.1733483, PMID: 32156153

[ref46] Singh G. B. Zhang Y. Boini K. M. Koka S. (2019). High mobility group box 1 mediates TMAO-induced endothelial dysfunction. Int. J. Mol. Sci. 20:3570. doi: 10.3390/ijms20143570, PMID: 31336567 PMC6678463

[ref47] Sun W. Debing D. Tongze F. Han Y. Li P. Hong J. (2022). Alterations of the gut microbiota in patients with severe chronic heart failure. Front. Microbiol. 12:813289. doi: 10.3389/fmicb.2021.813289, PMID: 35173696 PMC8843083

[ref48] Sun X. Jiao X. Ma Y. Liu Y. Zhang L. He Y. . (2016). Trimethylamine N-oxide induces inflammation and endothelial dysfunction in human umbilical vein endothelial cells via activating ROS-TXNIP-NLRP3 Inflammasome. Biochem. Biophys. Res. Commun. 481, 63–70. doi: 10.1016/j.bbrc.2016.11.017, PMID: 27833015

[ref49] Suzuki T. Heaney L. M. Bhandari S. S. Jones D. J. L. Ng L. L. (2016). Trimethylamine *N* -oxide and prognosis in acute heart failure. Heart 102, 841–848. doi: 10.1136/heartjnl-2015-308826, PMID: 26869641

[ref50] Swanson K. S. Gibson G. R. Hutkins R. Reimer R. A. Reid G. Verbeke K. . (2020). The international scientific Association for Probiotics and Prebiotics (ISAPP) consensus statement on the definition and scope of Synbiotics. Nat. Rev. Gastroenterol. Hepatol. 17, 687–701. doi: 10.1038/s41575-020-0344-2, PMID: 32826966 PMC7581511

[ref51] Tang W. H. W. Wang Z. Fan Y. Levison B. Hazen J. E. Donahue L. M. . (2014). Prognostic value of elevated levels of intestinal microbe-generated metabolite trimethylamine-N-oxide in patients with heart failure. J. Am. Coll. Cardiol. 64, 1908–1914. doi: 10.1016/j.jacc.2014.02.617, PMID: 25444145 PMC4254529

[ref52] Tatusov R. L. Fedorova N. D. Jackson J. D. Jacobs A. R. Kiryutin B. Koonin E. V. . (2003). The COG database: An updated version includes eukaryotes. BMC Bioinform 4:41. doi: 10.1186/1471-2105-4-41, PMID: 12969510 PMC222959

[ref53] Trøseid M. Ueland T. Hov J. R. Svardal A. Gregersen I. Dahl C. P. . (2015). Microbiota-dependent metabolite trimethylamine-N-oxide is associated with disease severity and survival of patients with chronic heart failure. J. Intern. Med. 277, 717–726. doi: 10.1111/joim.12328, PMID: 25382824

[ref54] Winkel P. Hilden J. Hansen J. F. Kastrup J. Kolmos H. J. Kjøller E. . (2015). Clarithromycin for stable coronary heart disease increases all-cause and cardiovascular mortality and cerebrovascular morbidity over 10years in the CLARICOR randomised, blinded clinical trial. Int. J. Cardiol. 182, 459–465. doi: 10.1016/j.ijcard.2015.01.020, PMID: 25602299

[ref55] Yang S. Li X. Yang F. Zhao R. Pan X. Liang J. . (2019). Gut microbiota-dependent marker TMAO in promoting cardiovascular disease: inflammation mechanism, clinical prognostic, and potential as a therapeutic target. Front. Pharmacol. 10:1360. doi: 10.3389/fphar.2019.01360, PMID: 31803054 PMC6877687

[ref56] Yao M.-E. Liao P.-D. Zhao X.-J. Wang L. (2020). Trimethylamine-N-oxide has prognostic value in coronary heart disease: a Meta-analysis and dose-response analysis. BMC Cardiovasc. Disord. 20:7. doi: 10.1186/s12872-019-01310-5, PMID: 31918665 PMC6953212

[ref57] Yazaki Y. Aizawa K. Israr M. Z. Negishi K. Salzano A. Saitoh Y. . (2020). Ethnic differences in Association of Outcomes with trimethylamine N-oxide in acute heart failure patients. ESC Heart Fail 7, 2373–2378. doi: 10.1002/ehf2.12777, PMID: 32598563 PMC7524106

[ref58] Yousefi B. Eslami M. Ghasemian A. Kokhaei P. Farrokhi A. S. Darabi N. (2019). Probiotics importance and their immunomodulatory properties. J. Cell. Physiol. 234, 8008–8018. doi: 10.1002/jcp.27559 30317594

[ref59] Zhang C. Yin A. Li H. Wang R. Guojun W. Shen J. . (2015). Dietary modulation of gut microbiota contributes to alleviation of both genetic and simple obesity in children. EBioMedicine 2, 968–984. doi: 10.1016/j.ebiom.2015.07.007, PMID: 26425705 PMC4563136

[ref60] Zhang J. Zhu P. Li S. Gao Y. Xing Y. (2023). From heart failure and kidney dysfunction to Cardiorenal syndrome: TMAO may be a bridge. Front. Pharmacol. 14:1291922. doi: 10.3389/fphar.2023.1291922, PMID: 38074146 PMC10703173

[ref61] Zhang J.-M. An J. (2007). Cytokines, inflammation, and pain. Int. Anesthesiol. Clin. 45, 27–37. doi: 10.1097/AIA.0b013e318034194e, PMID: 17426506 PMC2785020

[ref62] Zheng Y. Li Y. Rimm E. B. Hu F. B. Albert C. M. Rexrode K. M. . (2016). Dietary phosphatidylcholine and risk of all-cause and cardiovascular-specific mortality among US women and men. Am. J. Clin. Nutr. 104, 173–180. doi: 10.3945/ajcn.116.131771, PMID: 27281307 PMC4919531

[ref63] Zheng Y. He J.-Q. (2022). Pathogenic mechanisms of trimethylamine N-oxide-induced atherosclerosis and cardiomyopathy. Curr. Vasc. Pharmacol. 20, 29–36. doi: 10.2174/1570161119666210812152802, PMID: 34387163 PMC9680907

[ref64] Zong X. Fan Q. Yang Q. Pan R. Zhuang L. Xi R. . (2022). Trimethyllysine, a trimethylamine N-oxide precursor, predicts the presence, severity, and prognosis of heart failure. Front Cardiovasc Med 9:907997. doi: 10.3389/fcvm.2022.907997, PMID: 36247428 PMC9558138

