# Supplementary material for: The association between the gut microbiota metabolite trimethylamine N-oxide and heart failure
Source: Front Microbiol. 2024 Sep 26;15:1440241. doi: 10.3389/fmicb.2024.1440241 (PMC11464299; doi:10.3389/fmicb.2024.1440241)
Supplement: Supplementary file 1 [file Table_1.DOCX]

Supplementary Materials

Supplemental Table 1 Newcastle–Ottawa quality assessment scale (NOS).

| Study (reference) | Representativeness of the exposed cohort | Selection of the non exposed cohort | Ascertainment of exposure | Outcome of interest was not present at start of study | Comparability of cohorts based on the design or analysis | Assessment of outcome | Follow-up long enough for outcomes to occur | Adequacy of follow up of cohorts | Quality scores |
| --- | --- | --- | --- | --- | --- | --- | --- | --- | --- |
| Hayashi 2018 | * | * | * | * | * | * | * | * | 8 |
| Yazaki 2020 | * | - | * | - | * | * | - | * | 5 |
| Tang 2014 | * | * | * | * | * | * | * | - | 7 |
| Emoto 2021 | * | * | * | * | * | - | * | * | 7 |
| Amrein 2022 | * | * | * | * | * | * | * | * | 8 |
| Zong 2022 | * | * | * | - | * | * | * | * | 7 |
| Trøseid 2019 | * | * | * | - | * | * | * | * | 7 |
| Dong 2016 | * | * | * | * | * | * | * | - | 7 |
| Kinugasa 2021 | * | * | * | - | * | * | * | * | 7 |

Supplemental Table 2 **Detailed literature search strategy (Pubmed, Embase).**

| **No.** | **Query** | **Results** | **Date** |
| --- | --- | --- | --- |
| **#5** | #4 AND (2015:py OR 2016:py OR 2017:py OR 2018:py OR 2019:py OR 2020:py OR 2021:py OR 2022:py OR 2023:py) | 69 | 25 Jan 2024 |
| **#4** | #3 AND 'human'/de AND 'Article'/it | 70 | 25 Jan 2024 |
| **#3** | #2 AND ([adult]/lim OR [aged]/lim) | 144 | 25 Jan 2024 |
| **#2** | #1 AND 'heart failure'/dm | 658 | 25 Jan 2024 |
| **#1** | 'gut microbiota'/exp OR 'gut microbiota' OR (('gut'/exp OR gut) AND ('microbiota'/exp OR microbiota)) | 132732 | 25 Jan 2024 |

| **Search number** | **Query** | **Sort By** | **Filters** | **Search Details** | **Results** | **Time** |
| --- | --- | --- | --- | --- | --- | --- |
| **5** | gut microbiota heart failure |  | Humans, Aged: 65+ years, Adult: 19+ years | (("gastrointestinal microbiome"[MeSH Terms] OR ("gastrointestinal"[All Fields] AND "microbiome"[All Fields]) OR "gastrointestinal microbiome"[All Fields] OR ("gut"[All Fields] AND "microbiota"[All Fields]) OR "gut microbiota"[All Fields]) AND ("heart failure"[MeSH Terms] OR ("heart"[All Fields] AND "failure"[All Fields]) OR "heart failure"[All Fields])) AND ((humans[Filter]) AND (aged[Filter] OR alladult[Filter])) | 52 | 06:51:56 |
| **13** | gut microbiota heart failure |  | Humans, Adult: 19+ years, from 2013 - 2024 | (("gastrointestinal microbiome"[MeSH Terms] OR ("gastrointestinal"[All Fields] AND "microbiome"[All Fields]) OR "gastrointestinal microbiome"[All Fields] OR ("gut"[All Fields] AND "microbiota"[All Fields]) OR "gut microbiota"[All Fields]) AND ("heart failure"[MeSH Terms] OR ("heart"[All Fields] AND "failure"[All Fields]) OR "heart failure"[All Fields])) AND ((humans[Filter]) AND (alladult[Filter]) AND (2013:2024[pdat])) | 52 | 06:51:53 |
| **3** | gut microbiota heart failure |  | Humans, from 2013 - 2024 | (("gastrointestinal microbiome"[MeSH Terms] OR ("gastrointestinal"[All Fields] AND "microbiome"[All Fields]) OR "gastrointestinal microbiome"[All Fields] OR ("gut"[All Fields] AND "microbiota"[All Fields]) OR "gut microbiota"[All Fields]) AND ("heart failure"[MeSH Terms] OR ("heart"[All Fields] AND "failure"[All Fields]) OR "heart failure"[All Fields])) AND ((humans[Filter]) AND (2013:2024[pdat])) | 278 | 06:51:46 |
| **2** | gut microbiota heart failure |  | from 2013 - 2024 | (("gastrointestinal microbiome"[MeSH Terms] OR ("gastrointestinal"[All Fields] AND "microbiome"[All Fields]) OR "gastrointestinal microbiome"[All Fields] OR ("gut"[All Fields] AND "microbiota"[All Fields]) OR "gut microbiota"[All Fields]) AND ("heart failure"[MeSH Terms] OR ("heart"[All Fields] AND "failure"[All Fields]) OR "heart failure"[All Fields])) AND (2013:2024[pdat]) | 434 | 06:51:33 |
| **1** | gut microbiota heart failure |  |  | ("gastrointestinal microbiome"[MeSH Terms] OR ("gastrointestinal"[All Fields] AND "microbiome"[All Fields]) OR "gastrointestinal microbiome"[All Fields] OR ("gut"[All Fields] AND "microbiota"[All Fields]) OR "gut microbiota"[All Fields]) AND ("heart failure"[MeSH Terms] OR ("heart"[All Fields] AND "failure"[All Fields]) OR "heart failure"[All Fields]) | 435 | 06:51:25 |

Supplemental Table 3 Characteristics of study population in the included studies.

|  | **Low TMAO** | **High TMAO** |
| --- | --- | --- |
| **Age** |  |  |
| Emoto | 70(60-82) | 79(63-87) |
| Tang | 64 ± 11 | 68 ± 10 |
| Kinugasa | 80 [71–83] | 81 [75–86] |
| Zong | 61.3 ± 11.7 | 62.7 ± 11.4 |
| Amrein | 68.0 [59.0, 76.0] | 71.0 [63.0, 78.0] |
| **BMI** |  |  |
| Kinugasa | 21.23 [19.03–23.44] | 21.03 [18.44–23.12] |
| Amrein | 27.1 [24.2, 30.4] | 27.7 [24.8, 31.6] |
| Zong | 25.2 ± 3.7 | 24.9 ± 3.8 |
| **Male sex** |  |  |
| Emoto | 7/9 | 7/13 |
| Tang | 174/424 | 250/424 |
| Kinugasa | 10/33 | 17/33 |
| Zong | 226 /319 | 239/319 |
| Amrein | 752/1248 | 381/478 |
| **Smoking** |  |  |
| Emoto | 5/9 | 6/13 |
| Zong | 115/319 | 134/319 |
| Amrein | 750/1248 | 320/478 |
| **HFrEF, <40%** |  |  |
| Emoto | 6/9 | 6/13 |
| **HFpEF, ≥50%** |  |  |
| Emoto | 3/9 | 7/13 |
| **LV ejection fraction %** |  |  |
| Tang | 35 (25–51) | 40 (25–50) |
| Zong | 51.9 ± 16.7 | 46.4 ± 16.4 |
| Kinugasa | 59.80 [55.00–66.00] | 59.10 [54.45–65.00] |
| Emoto | 38 (20–59) | 43 (28–58) |
| Amrein | 60.0 [54.0, 62.5] | 55.0 [45.0, 60.0] |
| Zong | 51.9 ± 16.7 | 46.4 ± 16.4 |
| **Comorbidities** |  |  |
| *Hypertension* |  |  |
| Emoto | 8/9 | 13/13 |
| Tang | 426/561 | 443/561 |
| Kinugasa | 60/73 | 57/73 |
| Amrein | 965/1248 | 410/478 |
| Zong | 177/319 | 203/319 |
| *Diabetes mellitus* |  |  |
| Emoto | 6/9 | 2/13 |
| Tang | 91/295 | 150/295 |
| Kinugasa | 26/73 | 33/73 |
| Zong | 88/319 | 94/319 |
| Amrein |  |  |
| *Dyslipidemia* |  |  |
| Emoto | 5/9 | 4/13 |
| Kinugasa | 35/73 | 30/73 |
| Zong | 50/319 | 50/319 |
| *Atrial ﬁbrillation* |  |  |
| Emoto | 6/9 | 9/13 |
| Kinugasa | 37/73 | 36/73 |
| **Biochemical measures** |  |  |
| *Creatinine, mg/dl* |  |  |
| Emoto | 0.97 (0.84–1.12) | 1.29 (0.99–1.87) |
| Zong | 82.2 ± 27.3 | 118.3 ± 120.5 |
| *eGFR, ml/mm/1.73 m2* |  |  |
| Emoto | 57 (47–69) | 41 (21–48) |
| Tang | 83 (70–93) | 60 (44–74) |
| Kinugasa | 55.57 [42.82–73.10] | 32.49 [24.23–45.58] |
| Zong | 81.3 ± 18.8 | 68.9 ± 24.5 |
| Amrein | 82.0 [63.6, 93.7] | 75.9 [55.2, 88.5] |
| *BNP, pg/ml* |  |  |
| Emoto | 240 (114–359) | 325 (146–435) |
| Tang | 226 (96–498) | 358 (150–907) |
| Kinugasa | 160.95 [72.28–301.48] | 164.00 [89.80–329.60] |
